# An Improved Analytical Solution for Process-Induced Residual Stresses and Deformations in Flat Composite Laminates Considering Thermo-Viscoelastic Effects

**DOI:** 10.3390/ma11122506

**Published:** 2018-12-10

**Authors:** Chao Liu, Yaoyao Shi

**Affiliations:** The Key Laboratory of Contemporary Design and Integrated Manufacturing Technology, Ministry of Education, Northwestern Polytechnical University, Xi’an 710072, China; shiyy@nwpu.edu.cn

**Keywords:** residual stresses, deformations, thermo-viscoelastic, analytical solution, finite element, experimental results

## Abstract

Dimensional control can be a major concern in the processing of composite structures. Compared to numerical models based on finite element methods, the analytical method can provide a faster prediction of process-induced residual stresses and deformations with a certain level of accuracy. It can explain the underlying mechanisms. In this paper, an improved analytical solution is proposed to consider thermo-viscoelastic effects on residual stresses and deformations of flat composite laminates during curing. First, an incremental differential equation is derived to describe the viscoelastic behavior of composite materials during curing. Afterward, the analytical solution is developed to solve the differential equation by assuming the solution at the current time, which is a linear combination of the corresponding Laplace equation solutions of all time. Moreover, the analytical solution is extended to investigate cure behavior of multilayer composite laminates during manufacturing. Good agreement between the analytical solution results and the experimental and finite element analysis (FEA) results validates the accuracy and effectiveness of the proposed method. Furthermore, the mechanism generating residual stresses and deformations for unsymmetrical composite laminates is investigated based on the proposed analytical solution.

## 1. Introduction

Due to the high specific strength and stiffness, the fiber-reinforced epoxy resin composite materials have been widely applied in many fields including aerospace, automobile, civil infrastructures, and ship industries. However, residual stresses are inevitable in the composite structures during the manufacturing process. These process-induced residual stresses may lead to undesirable shape distortions when the cured components are released from the mold and such distortions are often large enough to render the part unserviceable. Moreover, residual stress during fabrication greatly decreases the fatigue life and dimensional accuracy of composite parts [1,2,3]. It is important to accurately predict the development of residual stresses and deformations during curing [4,5,6].

Many factors may govern the evolution of the residual stresses and deformation of composite structures during the fabrication process. The main factors include tool-part interaction, the mismatch of thermal and chemical properties of constitutive materials, and material degradation or viscoelastic effects during curing [1,7,8]. Numerous investigations regarding the effects of these previously mentioned factors on the process-induced residual stresses and deformations have been implemented by using analytical models and numerical methods based on finite element analysis [9,10,11,12,13]. 

The analytical method can provide quick prediction of residual stresses and deformations with a certain level of accuracy and explain the underlying mechanisms. Furthermore, the analytical method can also be used to validate the FE results for the simple cases where analytical solution can provide accurate results. Consequently, the analytical method has been increasingly applied in the investigations of cure behavior of composite structures during manufacturing [14,15,16,17,18,19,20,21,22,23]. Nelson and Cairns proposed a simple equation to predict the spring-in of the curved plate during the cooling-down stage based on thermo-elastic theory [14]. Radford et al. [15] modified this analytical model by taking into account the effects of chemical shrinkage of the resin on the spring-in. Moreover, a modified analytical model has been proposed to consider the effects of part thickness on the spring-in of C-shaped curved composite parts based on path-dependent constitutive models by Wisnom et al. [16]. The tool-part interactions have important influence on the residual stresses and deformations of composite structures, which have been considerably investigated by the experimental and finite element methods [4,7,24,25]. However, the effects of tool-part interactions on the residual stresses and deformations of composite structures have not been taken into account in the previously mentioned analytical models [4,5,6,7,8,9,10,11,12,13,14,15,16]. Consequently, a simple analytical model has been proposed to regard effects of the tool-part interactions on the cure-induced residual stresses and deformation of flat composite laminates by Twigg et al. [17]. The deformations of flat composite laminates obtained by this presented analytical solution align well with the experimental results. However, the dependence of residual stresses on material properties has not been captured by this model. Consequently, Arafath A et al. [19,20,21] presented a set of more accurate analytical models to predict the effects of tool-part interactions on the distribution of residual stresses and deformations for flat and curved composite laminates. The predictions of residual stresses and deformations obtained by these previously mentioned analytical models agree well with the numerical results and measured results. However, the viscoelastic effects of resin on the residual stresses and deformations of composite laminates during curing have not been taken into consideration in any of these previously mentioned models. 

In many recent studies, the viscoelastic effects of the resin regarding the cure-behavior of composite laminates have been considered through finite element analysis [26,27,28,29,30,31,32,33,34,35]. However, the finite element analysis is usually very time-consuming and it is difficult to gain any insight into the basic mechanism generating the residual stresses and distortions of composite parts during curing. In this paper, an improved analytical model is proposed by taking into account the viscoelastic effects of epoxy resin for the development of residual stresses and distortions of flat composite laminate during the manufacturing process. In the proposed model, the time-temperature superposition thermo-viscoelastic theory is introduced in this paper to include the viscoelastic effects of resin during the curing process. Moreover, the Timoshenko’s beam theory is adopted to describe the mechanical behavior of the composite layers or the tool-part interactions. An incremental formulation of the governing equation for the process-induced residual stresses and distortions with the viscoelastic effects is then established. Furthermore, a novel iterative strategy is proposed to solve the governing equation. To validate the accuracy and efficiency, a set of simulations are implemented based on the proposed model and finite element analysis. The corresponding FEA code was developed and incorporated into ANSYS with a User MAT subroutine. The simulation results obtained by the proposed model are compared to both the finite element results and experimental results.

## 2. Theoretical Development of the Analytical Solution with Thermo-Viscoelastic Effects

In this section, the proposed analytical solution with thermo-viscoelastic effects on the development of process-induced residual stresses and deformations is formulated in detail. An incremental formulation of the governing differential equation is first derived based on the Timoshenko’s beam theory and viscoelastic constitutive equation to describe the viscoelastic mechanical behavior of a unidirectional composite laminate during the curing process. Second, the analytical solution of the governing differential equation is developed. Third, the analytical solution of the residual stresses and deformations for multilayer composite laminates is derived based on the proposed analytical solution for unidirectional composite laminate. At last, the method to calculate the deformation of the composite laminates is given.

### 2.1. The Governing Differential Equation

**Figure 1:**
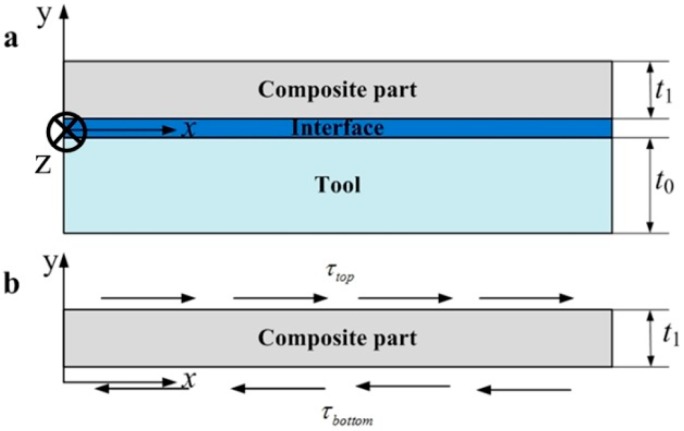
Schematic of a flat composite part on a solid tool. (**a**) Schematic of a composite part under sliding friction conditions due to tool expansion. (**b**) Schematic of a beam under applied shear tractions on the top and bottom surfaces.

The proposed analytical solution is based on the further improvement of the analytical solution proposed by Arafath A et al. [19,20,21]. Consequently, the same simplifications are adopted, which are described below:

(1) The Poisson’ effects are neglected.
(2) At the symmetrical y-z plane of the composite laminates shown in Figure 1a, the axial displacement equals zero.
(3) At the end of the composite laminates in the axial direction, the axial stresses are assumed to be zero.

As shown in Figure 1a, a composite ply would be stretched due to the tool expansion or the interactions of adjacent composite plies, which is a classical beam undergoing tractions at the top and bottom surfaces shown in Figure 1b. According to the Timoshenko’s beam theory [36], the equilibrium equation in the x direction in the absence of body forces at the time kΔt during the curing process can be given below:

(1) $$\frac{\partial \Delta \sigma_{xx}^{k}}{\partial x}+\frac{\partial \Delta \tau_{xy}^{k}}{\partial y}=0$$

where $\Delta \sigma_{xx}^{k}$ and $\Delta \tau_{xy}^{k}$ are the incremental axial stress and shear stress at time kΔt, respectively.

According to the integral form of polymer viscoelastic behavior and characteristics of thermo-rheologically simple materials, both the relaxed function and the constitutive relationship can be simplified to decouple the temperature effect using the time-temperature superposition (TTS) principle. A three dimensional (3D) incremental viscoelastic constitutive equation can be written as the equation below [27,28,29,30,31,32,33,34,35].

(2) $$\Delta \sigma^{k}=\sum_{i=1}^{w}\left(A_{2i}^{k}-1\right)\sigma^{k-1}+C^{*}\Delta \varepsilon_{eff}^{k}$$

where $\Delta \sigma^{k}$ and $\sigma^{k-1}$ are the incremental stress vector and stress vector at time kΔt and (k−1)Δt, respectively. w is the total number of the Maxwell elements. i is the serial number of each Maxwell element. $C^{*}$ is the time-dependent consistent tangent stiffness matrix at time kΔt, which can be defined by the equation below.

(3) $$C^{*}=C^{\infty }+\sum_{i=1}^{w}A_{1i}^{k}\omega_{i}\left(C^{u}-C^{\infty }\right)$$

where $C^{u}$ and $C^{\infty }$ are the fully unrelaxed and fully relaxed stiffness matrix, respectively. $\omega_{i}$ is the weigh factors for the ith Maxwell element. The stiffness $A_{1i}^{k}$ coefficient and stress relaxation coefficient $A_{2i}^{k}$ at time kΔt can be obtained as:

(4) $$A_{1i}^{k}=\frac{a_{T}\tau_{i}}{\Delta t}\left(1-exp\left(-\frac{\Delta \xi^{k}}{\tau_{i}}\right)\right),A_{2i}^{k}=exp\left(-\frac{\Delta \xi^{k}}{\tau_{i}}\right)$$

where $a_{T}$ denotes the shift factor as the function of degree of cure and temperature. $\tau_{i}$ is the discrete stress relaxation time for the ith Maxwell element. $\Delta \xi^{k}$ is the incremental reduced time at current time kΔt and can be calculated by $\Delta \xi^{k}=\Delta t/a_{T}$.

Without the Poisson’s effects, the incremental viscoelastic constitutive relationship between stress and strain at time kΔt can be written by the formulas below.

(5) $$\begin{array}{l} \Delta \sigma_{xx}^{k}=\sum_{i=1}^{w}\left(A_{2i}^{k}-1\right)\sigma_{xyi}^{k-1}+E_{xx}^{k}\Delta u_{x}^{k}\\ \Delta \tau_{xy}^{k}=\sum_{i=1}^{w}\left(A_{2i}^{k}-1\right)\tau_{xyi}^{k-1}+G_{xy}^{k}\Delta u_{y}^{k} \end{array}$$

where $\sigma_{xyi}^{k-1}$ and $\tau_{xyi}^{k-1}$ are the stress and shear stress of the ith Maxwell element at time (k−1)Δt, respectively. $\Delta u_{x}^{k}$ and $\Delta u_{y}^{k}$ are the first derivatives of axial mechanical displacement increment kΔt at time t with respect to the x and y, respectively. $E_{xx}^{k}$ and $G_{xy}^{k}$ denote the time-dependent consistent tangent axial and shear modulus at time kΔt, respectively. They can be obtained by using the equation below.

(6) $$E_{xx}^{k}=E_{xx}^{\infty }+\sum_{i=1}^{w}A_{1i}^{k}\omega_{i}\left(E_{xx}^{u}-E_{xx}^{\infty }\right),G_{xy}^{k}=G_{xy}^{\infty }+\sum_{i=1}^{w}A_{1i}^{k}\omega_{i}\left(G_{xy}^{u}-G_{xy}^{\infty }\right)$$

where $E_{xx}^{\infty }$ and $G_{xy}^{\infty }$ are the fully relaxed axial and shear modulus, respectively. $E_{xx}^{u}$ and $G_{xy}^{u}$ are the fully unrelaxed axial and shear modulus, respectively.

Substituting Equations (5) and (6) into Equation (1) and, after some rearrangements, the governing equation can be written by using the equation below.

(7) $$E_{xx}^{k}\Delta u_{xx}^{k}+G_{xy}^{k}\Delta u_{yy}^{k}=\Delta 1+\Delta 2$$

where $\Delta u_{xx}^{k}$ and $\Delta u_{yy}^{k}$ are the second derivative of axial displacement increment $\Delta u^{k}$ at time kΔt with respect to the x and y, respectively, and the parameters Δ1,Δ2 can be obtained by using the equations below.

(8) $$\begin{array}{l} \Delta 1=\sum_{i=1}^{w}\left(1-A_{2i}^{k}\right)E_{xxi}\left(A_{1i}^{k-1}\Delta u_{xx}^{k-1}+\sum_{p=1}^{\left(k-1\right)-1}\prod_{m=1}^{\left(k-1\right)-p}A_{2i}^{\left(k-1\right)-\left(m-1\right)}A_{1i}^{p}\Delta u_{xx}^{p}\right)\\ \Delta 2=\sum_{i=1}^{w}\left(1-A_{2i}^{k}\right)G_{xyi}\left(A_{1i}^{k-1}\Delta u_{yy}^{k-1}+\sum_{p=1}^{\left(k-1\right)-1}\prod_{m=1}^{\left(k-1\right)-p}A_{2i}^{\left(k-1\right)-\left(m-1\right)}A_{1i}^{p}\Delta u_{yy}^{p}\right) \end{array}$$

where $E_{xxi}$ and $G_{xyi}$ are the unrelaxed axial and shear modulus of the ith Maxwell element, respectively. They can be calculated by $E_{xxi}=\omega_{i}\left(E_{xx}^{u}-E_{xx}^{\infty }\right)$ and $G_{xyi}=\omega_{i}\left(G_{xy}^{u}-G_{xy}^{\infty }\right)$, respectively.

### 2.2. The Solution of the Governing Differential Equation

It can be seen that the governing Equation (7) is a second order non-homogeneous linear ordinary differential equation with constant coefficients. Usually, it is difficult to directly obtain the closed form solution for the governing Equation (7). However, there would be little viscoelastic effects when the material is in the fully-unrelaxed or fully relaxed state where both $A_{1i}^{k-1}$ and $A_{2i}^{k}$ equal to one or zero. Therefore, the previously mentioned parameters Δ1 and Δ2 would be equal to zero and Equation (7) would become the Laplace equation, which can be written by using the equation below.

(9) $$E_{xx}^{k}\Delta u_{xx}^{k}+G_{xy}^{k}\Delta u_{yy}^{k}=0$$

The Laplace Equation (9) can be directly solved by the method of separation of variables [19,20,21]. The solution of Equation (8) can be given as the equation below.

(10) $$\Delta u\left(k\Delta t\right)=\sum_{n=1}^{\infty }sin\left(k_{n}x\right)\left(A_{n}^{k}exp\left(\beta_{n}^{k}y\right)+B_{n}^{k}exp\left(-\beta_{n}^{k}y\right)\right)+\Delta \varepsilon_{the\text{-}chem}^{k}x$$

where $A_{n}^{k}$ and $B_{n}^{k}$ are the unknown constants and can be found from the boundary conditions at the interfaces. $\Delta \varepsilon_{the\text{-}chem}^{k}$ is the axial free thermal-chemical strain increment at time kΔt and can be calculated by $\Delta \varepsilon_{the\text{-}chem}^{kj}=\Delta T\alpha_{CTE}+\Delta C\gamma_{CSE}$.$\alpha_{CTE}$ and $\gamma_{CSE}$ are the coefficient of thermal expansion and the coefficient of shrinkage expansion, respectively. ΔT and ΔC are the temperature increment and cure degree increment. The characteristic constant $\beta_{n}^{k}$ is time-dependent and can be calculated by the equation below.

(11) $$\begin{array}{l} \beta_{n}^{k}=k_{n}\sqrt{\frac{E_{xx}^{k}}{G_{xy}^{k}}}\\ k_{n}=\left(2n-1\right)\pi /2l,n=1,2,3\ldots \end{array}$$

where l is half of the length of the laminate in the x direction.

It can be seen from Equation (7) that the axial displacement increment $\Delta u^{k}$ at time kΔt is related to the axial displacement increment of all the previous (k − 1) time steps. In order to obtain the solution of Equation (7), the time jΔt is defined by assuming that the material transforms from the fully relaxed state into a viscoelastic solid state. Readers can refer to Reference [34] for the method to calculate the time jΔt. For the fully relaxed state, the mechanical behavior of material can be described by the Laplace Equation (9) so that the solution at the time (j−1)Δt still conforms to the formulation as Equation (10). According to superposition principle of a differential equation with constant coefficients, it can be inferred that the solution of Equation (9) at time jΔt can be a linear combination of solutions of the corresponding Laplace equations at time (j−1)Δt and jΔt, which can be written as:

(12) $$\Delta u^{j}=\Delta u\left(j\Delta t\right)+\Delta u\left(\left(j-1\right)\Delta t\right)$$

To keep the balance on both sides of the equation, the non-homogeneous solution of equation $\Delta u^{k}$ must contain all the homogeneous solutions Δu(qΔt) of all the previous time steps. Since the parameters Δ1 and Δ2 are a linear combination of $\Delta u^{q}$, it can be inferred that the non-homogeneous solution of equation $\Delta u^{k}$ at time kΔt can be written as a linear combination of the homogeneous solution Δu(qΔt) of all time.

(13) $$\Delta u^{k}=\sum_{q=1}^{k}a_{k,q}\Delta u\left(q\Delta t\right)\left(q=1\cdots k\right)$$

Substituting Equation (13) into Equation (7) yields the coefficient $a_{k,q}$ below.

(14) $$a_{k,q}=\left\{ \begin{array}{l} 0,\left(G_{xy}^{k}-E_{xx}^{k}/c_{q}^{2}\right)=0\\ \sum_{i=1}^{w}\left(1-A_{{}_{2i}}^{k}\right)d_{i}^{k-1,q}\left(G_{xyi}-E_{xxi}/c_{q}^{2}\right)/\left(G_{xy}^{k}-E_{xx}^{k}/c_{q}^{2}\right),q<k-1\;and\;\left(G_{xy}^{k}-E_{xx}^{k}/c_{q}^{2}\right)\neq 0\\ \sum_{i=1}^{w}\left(1-A_{{}_{2i}}^{k}\right)A_{1i}^{k-1}\left(G_{xyi}-E_{xxi}/c_{k-1}^{2}\right)/\left(G_{xy}^{k}-E_{xx}^{k}/c_{k-1}^{2}\right),q=k-1\;and\;\left(G_{xy}^{k}-E_{xx}^{k}/c_{k-1}^{2}\right)\neq 0\\ 1,q=k \end{array}\right.$$

where $c_{q}$ is a time-dependent characteristic parameter and can be obtained by $c_{q}=\sqrt{E_{xx}^{q}/G_{xy}^{q}}$. The coefficient $d_{i}^{k-1,q}$ can be iteratively solved using the equation below.

(15) $$d_{i}^{k-1,q}=A_{2i}^{k-1}d_{i}^{k-2,q}+A_{1i}^{k-1}a_{k-1,q}$$

It can be seen that the coefficient $a_{k,q}$ is independent of the unknowns $A_{n}^{k}$ and $B_{n}^{k}$ and can be directly solved based on the calculation of material parameters of composite laminates during the curing process. The detailed derivation of the coefficient $a_{k,q}$ is shown in the Appendix.

### 2.3. The Solution of Residual Stresses for Multi-Layers Composite Laminates

**Figure 2:**
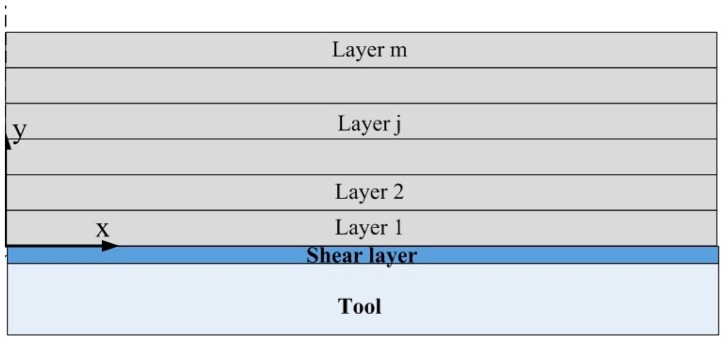
Model of the multi-layers composite laminates.

It is well known that the composite laminates are generally composed of laminate with different fiber orientations cured on a solid tool in autoclave, which is shown in Figure 2. To predict accurately the cure-induced residual stresses of multi-layers composite laminates, both the interactions of different composite layers and the tool-part interactions are considered in this scenario.

Formulation of stress and displacement

For the composite part:

From Equations (4)–(6) and (13)–(15), the stresses and displacement with viscoelastic effects of each composite layer can be determined by the equations below.

(16) $$\begin{array}{l} \Delta u^{k,j}=\sum_{q=1}^{k}a_{k,q}^{j}\Delta u^{j}\left(q\Delta t\right)+\Delta \varepsilon_{the\text{-}chem}^{kj}x\\ \Delta \sigma_{xx}^{k,j}=E_{xx}^{k,j}\sum_{q=1}^{k}a_{k,q}^{j}\Delta u^{j}{\left(q\Delta t\right)}_{x}+\sum_{i=1}^{w}\left(A_{2i}^{k}-1\right)E_{xxi}^{j}(\left(\sum_{q=1}^{k-2}d_{i,j}^{k-1,q}\Delta u^{j}{\left(q\Delta t\right)}_{x}\right)+A_{1i}^{k-1}\Delta u^{j}{\left(q\Delta t\right)}_{x})\\ \Delta \tau_{xy}^{k,j}=G_{xy}^{k,j}\sum_{q=1}^{k}a_{k,q}^{j}\Delta u^{j}{\left(q\Delta t\right)}_{y}+\sum_{i=1}^{w}\left(A_{2i}^{k}-1\right)G_{xyi}^{j}(\left(\sum_{q=1}^{k-2}d_{i,j}^{k-1,q}\Delta u^{j}{\left(q\Delta t\right)}_{y}\right)+A_{1i}^{k-1}\Delta u^{j}{\left(q\Delta t\right)}_{y}) \end{array}$$

where $\Delta u^{j}\left(q\Delta t\right)$ is the corresponding Laplace solution of the jth layer at time qΔt, which can be calculated by using the equation below.

(17) $$\Delta u^{j}\left(q\Delta t\right)=\sum_{n=1}^{\infty }sin\left(k_{n}x\right)\left(A_{n}^{q,j}exp\left(\beta_{n}^{q,j}y_{j}\right)+B_{n}^{q,j}exp\left(-\beta_{n}^{q,j}y_{j}\right)\right)$$

where $A_{n}^{q,j}$ and $B_{n}^{q,j}$ are unknowns associated with the jth layer.

For the tool:

As the tool is usually made of metallic materials, the relationship between the stress and strain can continually be described by the elastic theory and written as Equation (18) below [19,20,21].

(18) $$\begin{array}{l} \Delta {\bar{u}}^{k,0}=\sum_{n=1}^{\infty }D_{1n}^{k}sin\left(k_{n}x\right)cosh\left(\beta^{0}\left(y+t_{0}\right)\right)+\Delta \varepsilon_{therm}^{0}x\\ \Delta \sigma^{k,0}=\sum_{n=1}^{\infty }D_{1n}^{k}k_{n}cos\left(k_{n}x\right)cosh\left(\beta^{0}\left(y+t_{0}\right)\right)\\ \Delta \tau^{k,0}=\sum_{n=1}^{\infty }G_{0}D_{1n}^{k}\beta^{0}cos\left(k_{n}x\right)sinh\left(\beta^{0}\left(y+t_{0}\right)\right) \end{array}$$

where $G_{0}$ and $t_{0}$ are the shear modulus and thickness of tool material.

Boundary conditions

According to the layerwise approach [37], the layer axial displacement and shear stress should be equal on the interface between the two neighbor plies, which means that, at a generic jth interface ($y=y_{j}$), there are:

(19) $$\Delta {\bar{u}}^{k,j}=\Delta {\bar{u}}^{k,j-1},\Delta \tau_{xx}^{k,j}=\Delta \tau_{xx}^{k,j-1}$$

It can be seen that Equation (18) constitutes 2(m − 1) equations for (m − 1) interfaces.

For a stress free boundary condition at the top surface, the shear stress $\Delta \tau_{xy}^{k,m}$ can be expressed as:

(20) $$\Delta \tau_{xy}^{k,m}=0$$

Polymer film and a release agent from the mold usually separate the composite parts during autoclave processing. A compliant interface layer with shear modulus $G_{s}$ and thickness $t_{s}$ has been proposed to describe the tool-part interactions during curing by Arafath et al. [19,20,21]. The interface layer is very thin and the only stress that it transfers is a shear stress $\Delta \tau_{s}$ at time kΔt across its thickness, which is adopted in this scenario and can be formulated as:

(21) $$\begin{array}{l} \Delta \tau_{s}=G_{s}\frac{\Delta {\bar{u}}^{k,1}-\Delta {\bar{u}}^{k,0}}{t_{s}}\\ {\Delta \tau_{1}|}_{y=0}={\Delta \tau_{0}|}_{y=0}=\Delta \tau_{s} \end{array}$$

where $\Delta {\bar{u}}^{k,1}$ and $\Delta {\bar{u}}^{k,0}$ are the longitudinal displacements of the part and tool at the position y=0. As Equation (19)–(21) contains 2m + 1 equations, the total 2m + 1 unknowns including the unknowns $A_{n}^{q,j}$ and $B_{n}^{q,j}$ of each composite layer and the unknowns $D_{1n}^{k}$ of the tool can be determined and then the stresses and displacement of each layer can be calculated.

### 2.4. Deformations Prediction

At the end of the curing process, the tool is removed and the deformations are calculated by the unbalanced moment due to axial stress distribution through thickness. The moment can be obtained by using the equations below.

(22) $$\begin{array}{l} {M\left(x\right)|}_{k\Delta t}={M\left(x\right)|}_{\left(k-1\right)\Delta t}+{\Delta M\left(x\right)|}_{k\Delta t}\\ {\Delta M\left(x\right)|}_{k\Delta t}=\int_{0}^{t_{2}}b\Delta \sigma_{xx}^{k}\left(y-\frac{h}{2}\right)dy=\sum_{l=1}^{m}\int_{\left(l-1\right)t_{2}/m}^{lt_{2}/m}b\Delta \sigma_{xx}^{k}\left(y-\frac{h}{2}\right)dy \end{array}$$

where b is the beam width and h is the thickness of composite laminates.

Then, the bending deformation can be calculated by the equation below.

(23) $$\frac{d^{2}v}{dx^{2}}=\frac{M\left(x\right)}{{\left(EI\right)}_{eff}}$$

where ${\left(EI\right)}_{eff}$ is the bending rigidity of the composite laminate at the end of the cure cycle.

**Figure 3:**
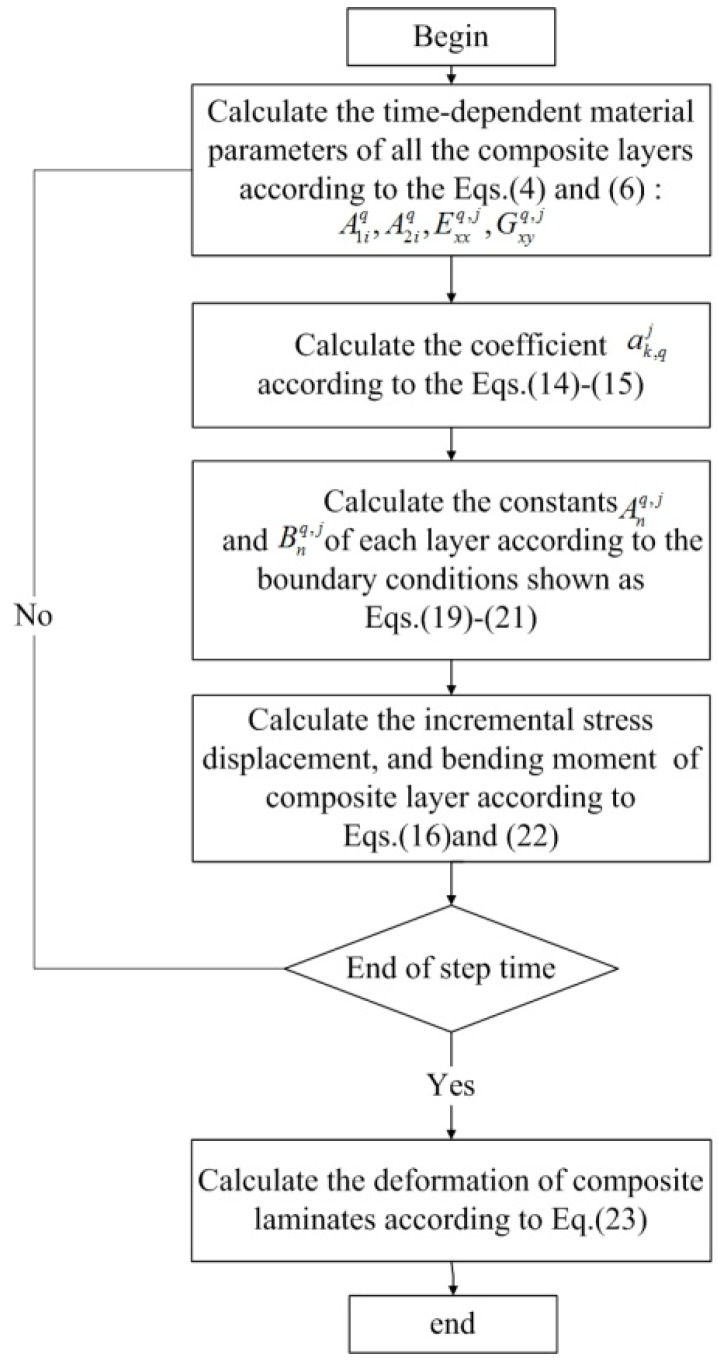
Flowcharts of calculation of residual stresses and deformations by the proposed analytical solution.

In summary, the process-induced residual stresses and deformations of composite components during curing can be solved by the flow chart shown in Figure 3.

## 3. Numerical Simulation and Experimental Investigation

### 3.1. Material Properties and MODEL

Both numerical simulation and experimental verification for the symmetrical and unsymmetrical composite laminates [0_2_/90_2_]s and [0_4_/90_4_] would be carried out in this paper to verify the efficiency and accuracy of the proposed analytical model and compared with previous studies. The illustrations of the materials and model are described as follows.

#### 3.1.1. Material Properties

The composite parts used in this study are made of graphite/epoxy (AS4/3501-06) unidirectional prepregs from Hexcel Composites, which has been used for thermo-viscoelastic analysis during the curing process in previous studies [25,26,27,28,29,30,31,32,33,34,35]. The viscoelastic material properties are listed in Table 1 and Table 2, respectively. It is assumed that the initial resin modulus in the viscous state is 5–6 orders of magnitude less than that of the fully cured resin, which has been verified by experimental results [19,20,21,22,23] and is adopted in this case.

**Figure 4:**
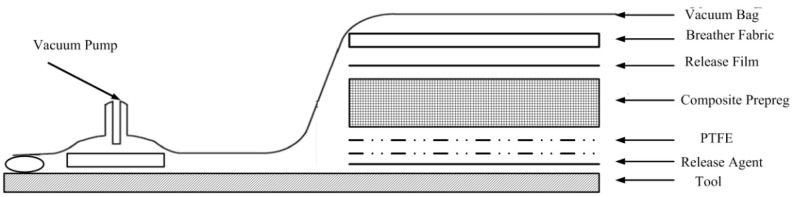
Vacuum bagging system for the autoclave process.

The composite specimen strips are generally manufactured by the autoclave assisting cure method, which is shown in Figure 4. It can be seen that a polymer release film and release agent separates the tool and the composite part during the curing process in the autoclave. During the curing process, the tool and composite part will interact with each other due to the mismatch of thermal-chemical properties between different materials. To improve the efficiency of modeling, the release film and the agent between the tool surface and the composite have been modeled as a shear layer, which is very thin, and the only stress that it transfers is a shear stress that is uniform across its thickness. The material properties of the tool and the shear layer are shown in Table 3 [19,20,21].

#### 3.1.2. Finite Element Model

**Figure 5:**
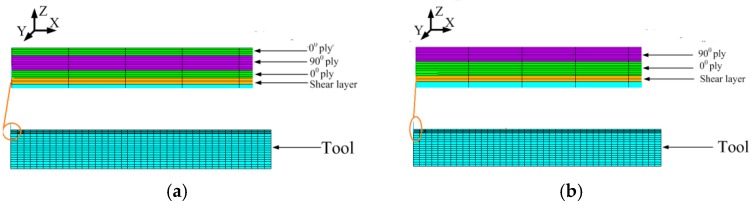
Finite element meshes. (**a**) Finite element meshes of symmetrical laminate with layup [0_2_/90_2_]_s_. (**b**) Finite element meshes of unsymmetrical laminate with layup [0_4_/90_4_].

The simulations are implemented based on both the proposed new analytical solution and the viscoelastic finite element analysis. The linear element solid 186 with 20 nodes is used in the FEA analysis in which the three-dimensional thermo-viscoelastic constitutive Equation (2) is incorporated into finite element software ANSYS as a user subroutine USERMAT to describe viscoelastic behavior of materials. Considering the symmetrical geometry and boundary conditions of the composite laminate, only 1/4 part of the specimen is modeled. The symmetrical constraints are applied in the X-Z and Y-Z symmetrical planes. The longitude and transverse directions are defined as X and Y, respectively. The thickness direction is defined as Z. Each ply contains 2 elements and 40 elements along x directions for the composite laminate. The shear layer ply is meshed into 2 elements through the thickness direction. The total number of nodes and elements are 9608 and 1320, respectively. The chemical shrinkage of the resin and cure thermal loads are treated as equivalent thermal loads and applied in the FEA model. The finite element analysis model is shown as Figure 5. In the finite element analysis by ANSYS, the cycle solution strategy is applied to solve the cure-induced residual stresses and deformations of the composite structure.

The thermo-chemical analyses should be developed to obtain the temperature and degree of cure distributions throughout the laminates [4,35]. However, the thickness of composite laminates used in this case is 1 mm, which is so thin that the cure degree and temperature field can be considered uniform. Thus, thermo-chemical analysis is not presented in this scenario.

### 3.2. Case 1: Numerical Simulation for Symmetrical Composite Laminates

In this section, numerical simulation for the process-induced stresses and deformations of composite laminates with [0_2_/90_2_]s layups cured on a solid tool is implemented based on the analytical solutions and finite element analysis. The recommended manufacturing cure cycle presented in Reference [35] in the autoclave consists of a first ramp of 2.5 °C/min up to 116 °C hold for 60 min, a second ramp of 2.5 °C/min up to 175 °C, which holds for 20 min, and a cool down of −2.5 °C /min up to 25 °C.

**Figure 6:**
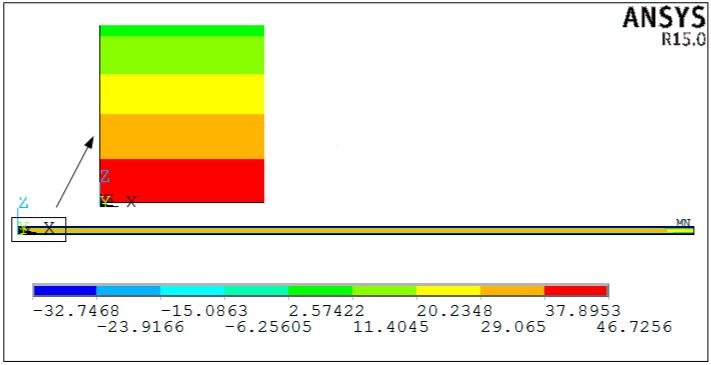
The stress distribution of symmetrical composite laminates obtained by viscoelastic FEA.

**Figure 7:**
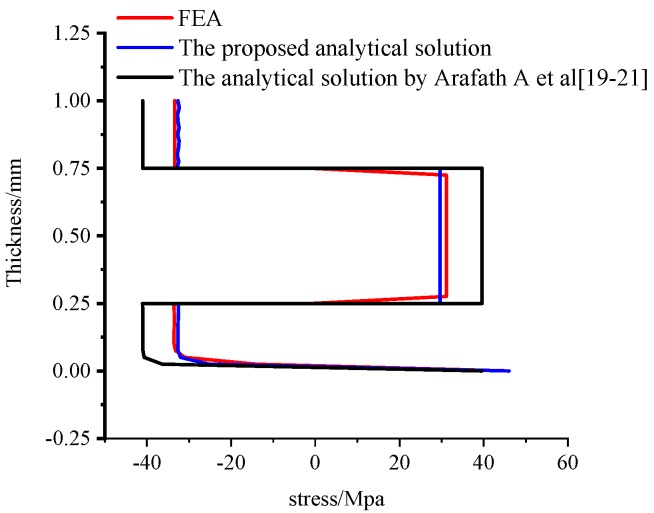
Comparison of a numerical and an analytical predicted stress distribution through-thickness of unsymmetrical composite laminates.

Figure 6 shows the axial stress distribution obtained by the viscoelastic finite element analysis. Figure 7 shows the comparison of the numerical predictions and analytical predictions of axial stress distribution through-thickness direction. The analytical predictions are both implemented by the proposed analytical solution and the analytical solution presented by Arafath A et al. [19,20,21], respectively. It can be seen that the results obtained by the proposed model show good agreement with the results of viscoelastic finite element analysis. The maximum difference of axial stresses between the proposed analytical method and the viscoelastic finite element analysis is 1.3 Mpa, which corresponds to a relative error rate of 3.8%. On the contrary, it can be observed that there are large differences between the residual stresses obtained by the analytical solution presented by Arafath A et al. [19,20,21] and the proposed analytical solution. The maximum difference of axial stresses is 11.8 Mpa, which corresponds to a relative error rate of 40.3%.

These simulation results show that the proposed analytical solution can deal well with the viscoelastic effects of the resin on the residual stresses of symmetrical composite laminates and is more accurate than the analytical solution presented by Arafath A et al. [19,20,21].

**Figure 8:**
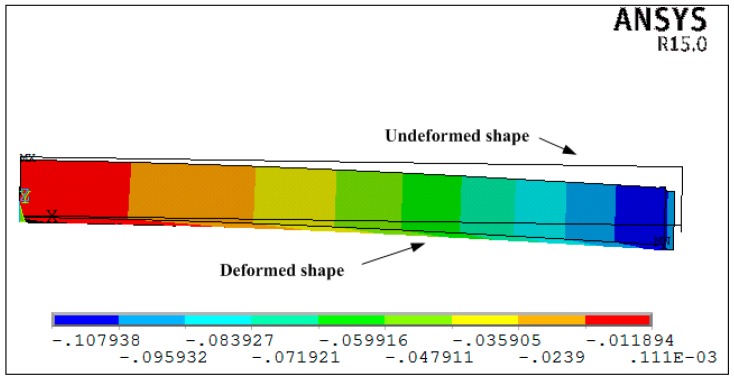
The deformation of symmetrical composite laminates predicted by viscoelastic FEA.

**Figure 9:**
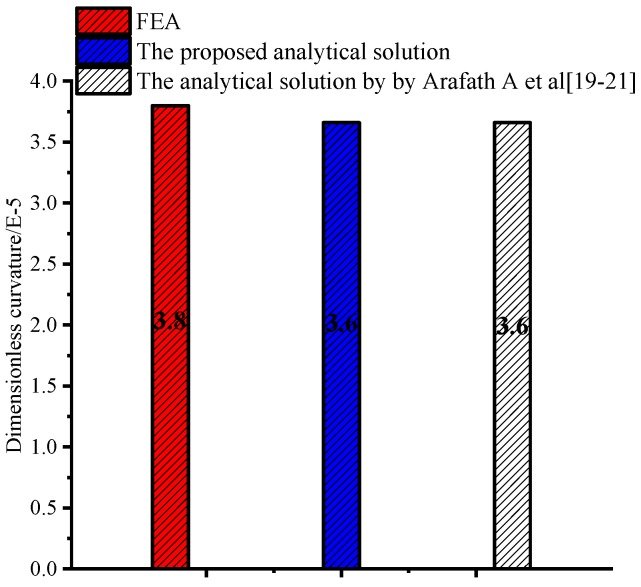
Comparison of numerical and analytical predicted dimensionless curvature of symmetrical composite laminates.

Figure 8 shows the prediction of the deformations obtained by the viscoelastic FEA. Figure 9 shows the prediction of the deformations of the composite laminates at the end of the cure process, which are also calculated by the previously mentioned methods. It can be observed that the results obtained by the proposed analytical solution and the analytical solution by Arafath A et al. [19,20,21] are in good agreement with the results obtained by viscoelastic finite element analysis. Moreover, it can be seen that the residual stresses change sharply from compression to tension in the 0°–90° interface. This can be explained by the composite laminates contracting due to chemical shrinkage of the resin and the cooling process when curing. As both the thermal and chemical shrinkage coefficients of 90° composite layers are larger than those of 0° composite layers, the 0° layers would be compressed and, thus, the 90° layers would be tensioned to keep balance. These results indicate that the proposed analytical solution can accurately predict the cure-induced deformations of symmetrical composite laminates compared with Arafath A et al. [19,20,21].

**Figure 10:**
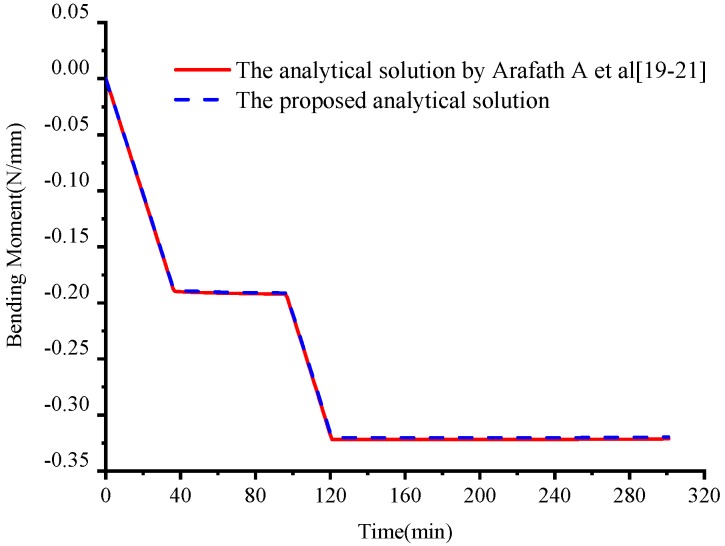
Comparison of the development of the bending moment by different analytical models of symmetrical composite laminates.

Figure 10 shows the comparison of development of the resultant bending moment during the curing process between the proposed analytical solution and the analytical solution presented by Arafath A et al. [19,20,21]. It can be seen that the bending moment mainly occurs at the initial stages of the curing process when the epoxy resin is in the viscous state and shear modulus is very low. In a viscous state, the matrix is in the fully-relaxed state so that there are no obvious viscoelastic effects. The proposed analytical solution would degenerate into the elastic solution, which is the same as the analytical solution proposed by Arafath A et al. [19,20,21]. Therefore, the bending moment obtained by the proposed model is the same as that obtained by the analytical model proposed by Arafath A et al. [19,20,21] in the viscous state before the gelation point. Since the stresses produced in the viscoelastic state are basically symmetrical for the x-z plane, there is almost no bending moment. Therefore, the deformation obtained by the proposed analytical solution agrees with that of the analytical solution presented by Arafath A et al. in the viscoelastic state after gelation.

At length, the efficiency of the proposed analytical solution in calculating the residual stress and deformations is compared to that of the viscoelastic finite element analysis and the analytical solution presented by Arafath A et al. [19,20,21]. It can be seen from Table 4 that, although the run-time of the proposed analytical model is slightly greater than the model proposed by Arafath A et al. [19,20,21], the proposed analytical model is still very efficient when compared to the FEA model. The run-time of the proposed analytical solution is less than 20 s in this case.

These previously mentioned results show that the proposed analytical solution is more accurate and efficient to predict the process-induced residual stresses and deformation of the symmetrical composite laminates compared to the solution presented by Arafath A et al. [19,20,21].

### 3.3. Case 2: Numerical and Experimental Investigation for Unsymmetrical Composites

#### 3.3.1. Illustration of the Model

**Figure 11:**
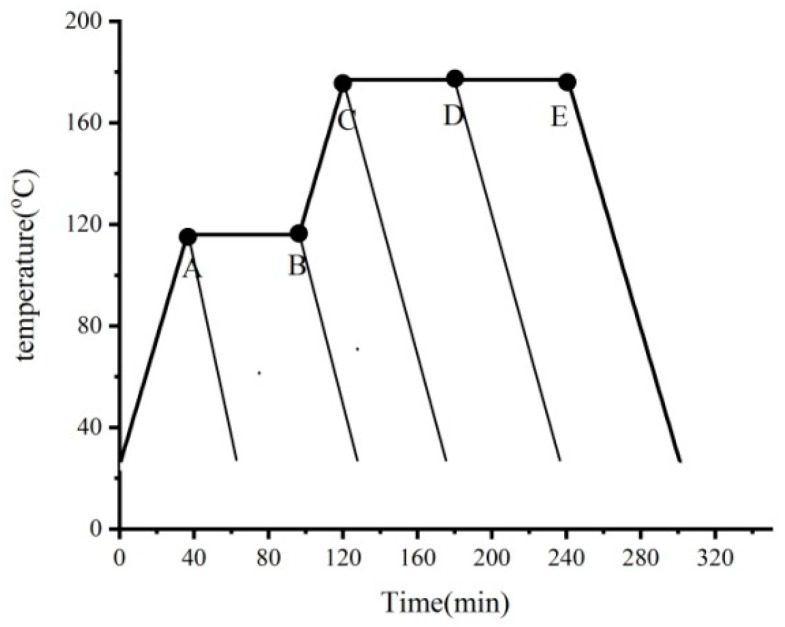
Interrupted cure cycles.

Compared to the investigations for the cure behavior of symmetrical composite laminates, there is limited knowledge on the development of residual stresses and deformation of the unsymmetrical composite laminates during curing. However, it does not mean that coupled unsymmetrical laminates are useless. They can be used during unique conditions [38,39,40,41]. Considering that this paper focuses on the viscoelastic effects of the matrix on the process-induced residual stresses and distortions, a set of specimen strips with [0_4_/90_4_] layup composite laminates, which has been experimentally investigated by Kim and Hahn in Reference [38] are taken as an example for simulations to further verify the accuracy and effectiveness of the proposed analytical model. In the study mentioned earlier [38], a set of unsymmetrical composite laminates made of graphite/epoxy (T300/3501-6) prepregs have been subjected to interrupted cure cycles shown in Figure 11. The heating and cooling rates are set to be approximately 3 °C/min. The unsymmetrical composite laminates are 152 mm long strips with a width of 25 mm. The assumption that materials properties of T300/3501-6 prepregs are the same as those of AS4/3501-6 prepregs in Reference [38] is adopted in the following investigations.

#### 3.3.2. Verification of the Accuracy and Effectiveness

**Figure 12:**
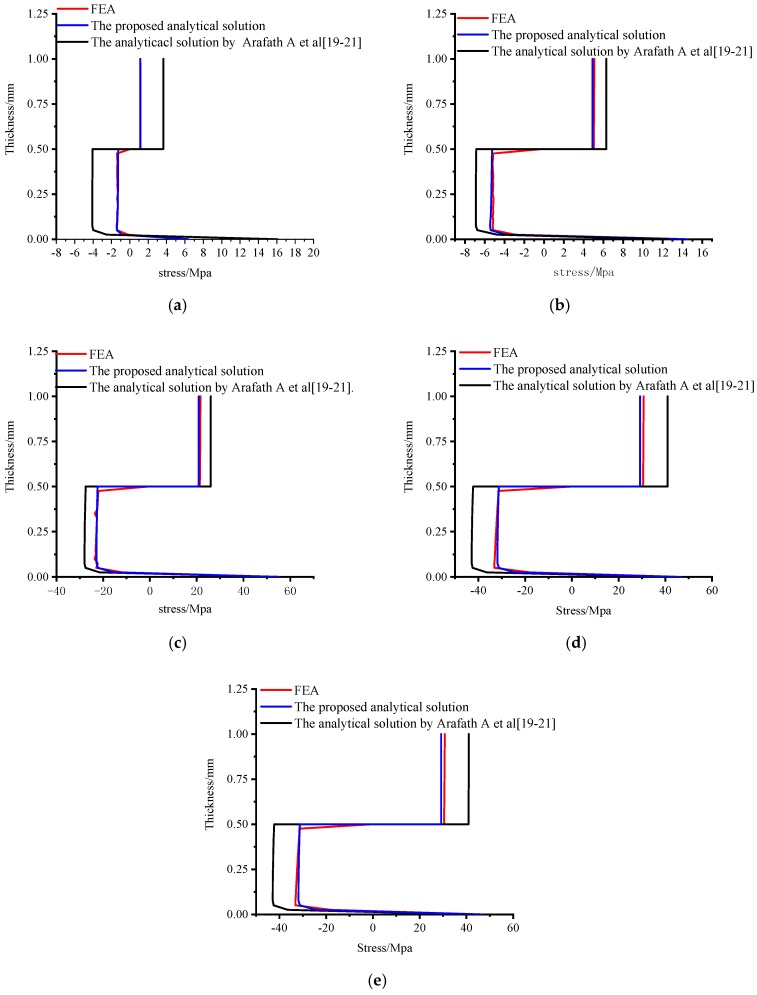
Comparison of numerical and analytical predicted stress distribution through-thickness of unsymmetrical composite laminates. (**a**) The axial stress distribution through-thickness direction under cure cycle A. (**b**) The axial stress distribution through-thickness direction under cure cycle B. (**c**) The axial stress distribution through-thickness direction under cure cycle C. (**d**) The axial stress distribution through-thickness direction under cure cycle D. (**e**) The axial stress distribution through-thickness direction under cure cycle E.

Figure 12a–e shows the comparison of numerical and analytical predicted axial stresses distribution of the central point of the unsymmetrical composite laminates through-thickness direction at the end of the cure cycles A–E. It can be observed that all of the axial stress under these five cure cycles A–E obtained by the proposed analytical solution agree well with those obtained by the viscoelastic finite element analysis. However, there are clear differences of the axial stress under these five cure cycles A–E between the results obtained by the proposed analytical solution and the analytical solution presented by Arafath A et al. [19,20,21].

**Figure 13:**
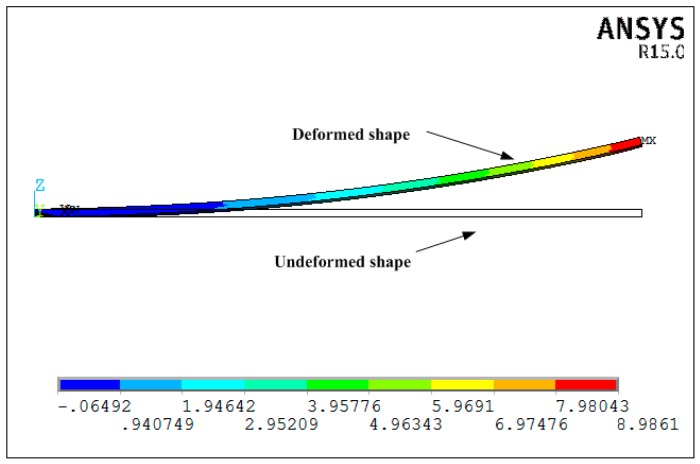
The deformation of unsymmetrical composite laminates under cure cycle E predicted by viscoelastic FEA.

**Figure 14:**
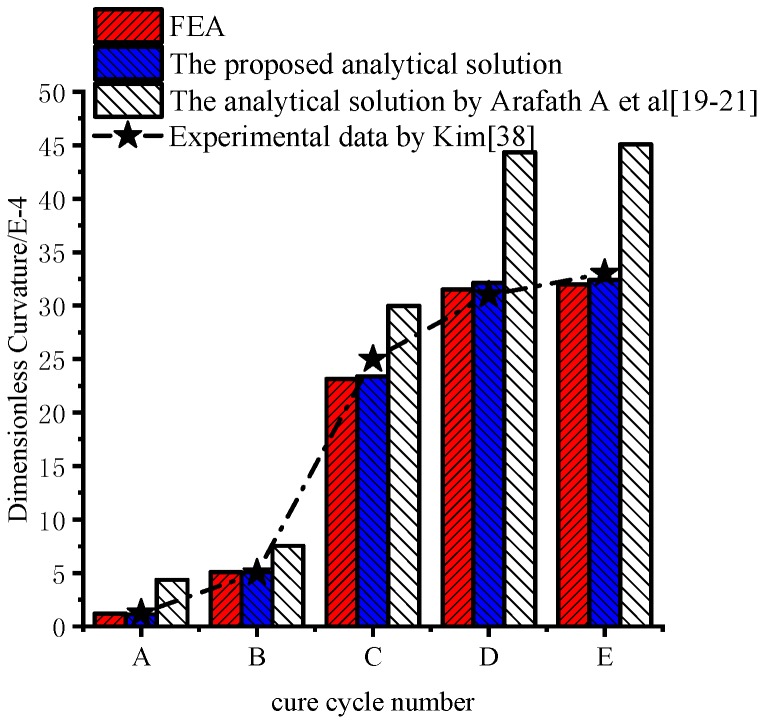
Comparison of experimental, analytical, and numerical predicted dimensionless curvature of unsymmetrical composite laminates.

Figure 13 shows the deformations of unsymmetrical composite laminates obtained by the viscoelastic FEA. The predicted dimensionless curvatures under the interrupted cure cycles A–E obtained by the proposed analytical solution are shown in Figure 14. The results are obtained by the analytical solution presented by Arafath A et al. [19,20,21] and the viscoelastic finite element analysis as well as the experimental data presented by Kim et al. [38], respectively. It can be seen that the predicted dimensionless curvatures under all cure cycles by the proposed analytical solution are in good agreement with both the experimental results and the results obtained by FEA with the viscoelastic constitutive law. However, all of the predicted values under all cure cycles obtained by the analytical solution presented by Arafath A et al. [19,20,21] are much larger than the predicted results of the proposed analytical solution and experimental values.

These previously mentioned results show that the proposed analytical solution can deal well with the viscoelastic effects of the polymer matrix and provide a more accurate prediction of the development of residual stresses and deformation of the unsymmetrical composite laminates during curing than the analytical solution presented by Arafath A et al. [19,20,21].

#### 3.3.3. The Further Investigations on the Cure Mechanism

It is well known that the residual stresses and deformation of composite laminates are influenced by many factors including tool-part interactions, thermal effects, chemical shrinkage of the resin, and stress relaxation of matrix. However, the mechanism concerning the effects of these above factors on the development of residual stresses and deformation for the unsymmetrical composite laminates during curing has not been clearly clarified. Therefore, a further investigation is implemented to gain insight into the generation mechanism of the residual stresses and deformation based on the proposed analytical solution.

#### 3.3.4. Effects of Chemical Shrinkage

**Figure 15:**
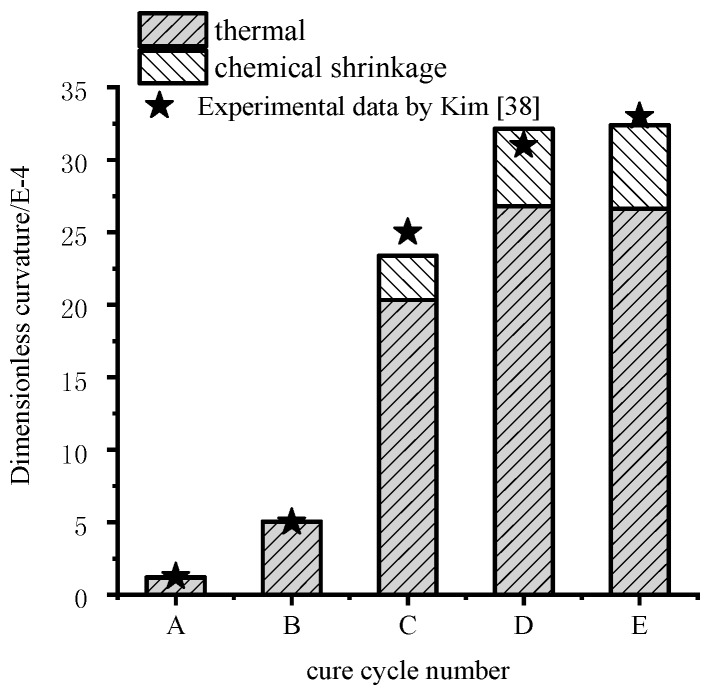
The effects of chemical shrinkage of the resin on curvature by the proposed analytical solution.

The dimensionless curvatures of unsymmetrical composite laminates under the cure cycles A–E are calculated based on the proposed analytical solution and are shown in Figure 15 in which both the cases with thermal effects alone and chemical shrinkage of the resin are implemented. It can be seen that the effects of chemical shrinkage of the resin on the curvature are dependent on the degree of cure and cure cycles.

Moreover, it can be seen from Figure 15 that the chemical shrinkage of the resin has no significant influences on the deformation of unsymmetrical composite laminates under cure cycle A and B. Moreover, there is a better agreement between measured and predicted values obtained by the proposed model considering the chemical shrinkage of the resin when compared to the cases with thermal effects alone after gelation. For the results obtained by the proposed model under cure cycle C, it can be seen that the chemical shrinkage of the resin accounts for about 8% of the final curvature. For the results obtained by the proposed model under cure cycles D and E, it can be seen that the chemical shrinkage of the resin accounts for about 17% of the final curvature. It is very close to the experimental results 20% for a non-symmetrical [0/90] lay-up composite laminate made of AS4/8852 prepregs by R. Akkerman et al. [39].

**Figure 16:**
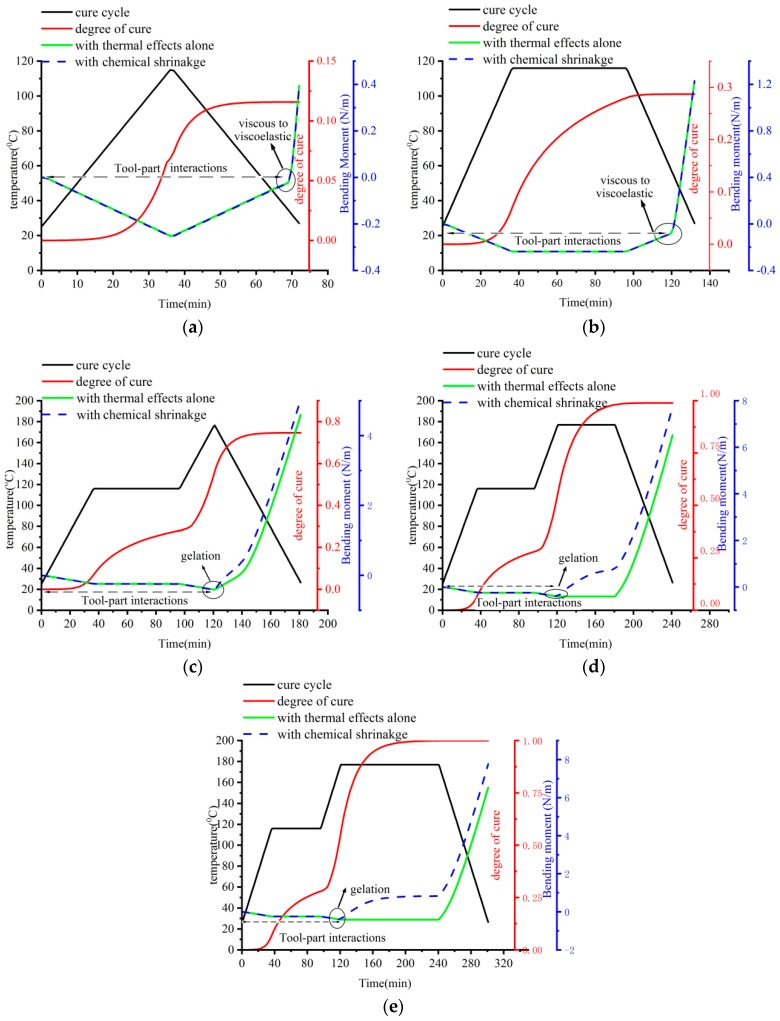
Comparison of the development of the bending moment between thermal effects and chemical shrinkage obtained by the proposed analytical solution. (**a**) The development of the bending moment under cure cycle A. (**b**) The development of the bending moment under cure cycle B. (**c**) The development of the bending moment under cure cycle C. (**d**) The development of the bending moment under cure cycle D. (**e**) The development of the bending moment under cure cycle E.

The bending moment at the end of curing determines the deformation of the composite laminates. To further investigate the mechanism of cured-deformation, the developments of bending moments under the cure cycles numbered A–E are calculated based on the proposed analytical solution and the solution presented by Arafath A et al. [19,20,21] shown in Figure 16 in which both cases with thermal effects alone and chemical shrinkage of the resin are implemented.

It can be seen from Figure 16a,b that there is no significant difference regarding the developments of the bending moment between the case with thermal effects alone and that with chemical shrinkage of the resin under the cure cycle A and B. This is similar to the results of dimensionless curvature shown in Figure 15. It can be explained by the fact that the modulus of the matrix is very low in the viscous state, which means no significant stress can be developed in the composite laminates. These negative bending moments can be caused by the shear interactions between tool and composite parts. In addition, it can be observed from Figure 16a,b that there are some positive bending moments during the end stage of the cool-down process, which can be considered to be caused by thermal mismatch of different composite layers. Both the degree of cure is very low so that the relaxation time of the resin system is very low. Therefore, only some small residual stresses have been formed at the end of the cooling process due to interactions of different composite layers. Furthermore, the degree of cure stops increasing at this stage and, therefore, there is no effect of the chemical shrinkage of the resin on the residual stresses and the bending moment under cure cycle A and B.

Moreover, it can be seen from Figure 16c–e that the cure shrinkage of the matrix has clear effects on the development of the bending moments after gelation. The degree of cure under the cure cycle is lower than those under the cure cycle D and E. Therefore, the effects of chemical shrinkage on the bending moment under the cure cycle C are smaller than those under cure cycles D and E. The effects of the chemical shrinkage of the final curvature under cure cycle C are lower than those under cure cycle E. The effects of chemical shrinkage on the final curvature under cure cycle C shown in Figure 15 are, thus, smaller than those under cure cycle D and E shown in Figure 15, respectively. In addition, it can be seen from Figure 16d,e that the effects of chemical shrinkage of the resin on the evolution of the bending moments begins from gelation to the end of the second dwelling under cure cycle D and E. The matrix has been almost fully cured at the end of cure cycles D and E so that the bending moments at the end of cure cycle D are nearly equal to those at the end of cure cycle E. Therefore, the effects of the chemical shrinkage of the final curvature under cure cycle D in Figure 15 are nearly the same as those under cure cycle E in Figure 15.

#### 3.3.5. Effects of Tool-Part Interactions

It can also be seen from Figure 16 that the bending moment due to the tool-part interactions is mainly formed at a viscous state before gelation and is very small when compared to those due to different composite layer interactions that occurred at a viscoelastic state after gelation. The tool-part interactions contribute to the bending moment and deformation by about 4.8 % at the end of cure cycle E. However, it can be seen from Figure 12 that there is a significant stress gradient close to the tool-part interface due to tool-part interactions for all the cases under cure cycles A–E. It can be, thus, concluded that the effects of tool-part interactions on the residual stresses cannot be neglected for the unsymmetrical composite laminates.

## 4. Conclusions

In this paper, an improved analytical solution considering the viscoelastic effects on the residual stresses and deformations of flat composite laminates has been established based on the modification of a previous analytical solution. Specifically speaking, the proposed analytical solution is derived based on the Timoshenko’s beam theory and the time-temperature superposition thermo-viscoelastic theory. The accuracy and effectiveness of the proposed analytical solution is verified through a comparison of results with both experiment and viscoelastic FEA for symmetrical and unsymmetrical composite laminates. The numerical simulation and experimental investigation results show that many important factors inducing the residual stresses and deformations such as tool-part interaction, thermal mismatch, chemical shrinkage, and viscoelastic effects of the resin can be accurately analyzed by the proposed analytical solution. In the numerical simulations and experimental investigations, some important findings are summarized below.

(1) The proposed analytical solution can deal well with the viscoelastic effects of the polymer matrix on the residual stresses and deformations during the curing process. The proposed analytical solution is more efficient and has a similar accuracy to 3-D viscoelastic finite element analysis.
(2) The prediction of deformation for symmetrical laminates with layups [0_2_/90_2_]s by the proposed analytical solution yields similar results with that of the analytical solution presented by Arafath A et al. [19,20,21]. However, the proposed analytical solution provides much better predictions of the residual stresses for the symmetrical composite laminates with layups [0_2_/90_2_]s.
(3) For the unsymmetrical composite laminates with layups [0_4_/90_4_], the proposed analytical solution can provide much better results of both residual stresses and deformation with an improvement of at least 41% compared with the results obtained by the analytical solution presented by Arafath A et al. [19,20,21].
(4) The effects of cure shrinkage of the polymer matrix on the residual stresses and deformation for unsymmetrical composite laminates depend on the degree of cure and cure cycles. There are almost no clear effects of cure shrinkage of polymer matrix on the residual stresses and deformation before gelation. Chemical shrinkage of the resin during the recommended cure cycle contributes to the residual stresses and curvature by about 17%.
(5) Apart from symmetrical composite laminates, tool-part interactions have little influence on the deformations of unsymmetrical composite laminates. Tool-part interactions contribute to the deformation of unsymmetrical composite laminates with a percentage of 4.8%. However, the effects of tool-part interactions on the residual stresses distribution of unsymmetrical composite laminates are similar to those of symmetrical composite laminates and cannot be neglected.

## Figures and Tables

**Table 1:** Constituent material properties of AS4 fiber and 3501-6 epoxy resin.

Property	AS4 Graphite Fiber	3501-6 Epoxy Resin
$E_{1}\left(GPa\right)$	207.0	3.2
$E_{2}=E_{3}\left(GPa\right)$	20.7	3.2
$G_{12}=G_{13}\left(GPa\right)$	27.6	1.19
$G_{23}\left(GPa\right)$	6.89	1.19
$v_{12}=v_{13}$	0.2	0.35
$v_{23}$	0.3	0.35
$\alpha_{1}^{th}(\mu \varepsilon /{}^{\circ}C)$	−0.9	57.6
$\alpha_{2}^{th}=\alpha_{3}^{th}(\mu \varepsilon /{}^{\circ}C)$	7.2	57.6
$\gamma_{1}=\gamma_{2}=\gamma_{3}\left(\mu \varepsilon \right)$	0	−16,950

**Table 2:** Relaxation times and weight factors at the reference degree of cure 0.98.

i	$\tau_{i}\left(\min\right)$	$\omega_{i}$
1	$2.922137\times 10^{1}$	0.0591334
2	$2.921437\times 10^{3}$	0.0661255
3	$1.82448\times 10^{5}$	0.0826896
4	$1.1031059\times 10^{7}$	0.112314
5	$2.8305395\times 10^{8}$	0.154121
6	$7.9432822\times 10^{9}$	0.2618288
7	$1.953424\times 10^{11}$	0.1835594
8	$3.3150756\times 10^{12}$	0.0486939
9	$4.9174856\times 10^{14}$	0.0252258
$a_{T}=10^{\left(-1.4exp\left(1/\left(\alpha -0.98\right)\right)-0.0712\right)\left(T-30\right)}$		

**Table 3:** Material properties of the aluminum tool and the shear layer.

Properties	Elastic Modulus E (GPa)	Shear Modulus G (GPa)	Poisson’s Ratio ν	Coefficient of Thermo Expansion α (με/°C)	Thickness (mm)
Aluminum tool	69.0	26	0.33	26.3	10
Shear layer	/	20 × 10^−6^	/	/	0.2

**Table 4:** Comparison of the required run-times for the analytical model and the FEA method.

Total Time Increments	Run Time(s)		
	The Analytical Model Proposed by References [19,20,21]	The Proposed Analytical Model	FEA with Viscoelastic Constitutive Law
75	0.26	3.24	380.54
135	0.49	5.91	732.91
180	0.82	9.54	1033.69
242	1.03	12.14	1356.72
302	1.18	12.69	1564.38

## Appendix A

In this section, the detailed derivation of the solution of the coefficient $a_{k,q}$ and $d_{i}^{k-1,q}$ are given. Substituting Equation (13) into Equation (8) and pursuing some rearrangements, the differential Equation (8) can be formulated as follows:

(A1) $$\begin{array}{l} a_{k,k}\sum_{n=1}^{\infty }\left(G_{xy}^{k}{\left(\beta_{n}^{k}\right)}^{2}-E_{xx}^{k}{\left(k_{n}\right)}^{2}\right)sin\left(k_{n}x\right)\left(A_{n}^{k}exp\left(\beta_{n}^{k}y\right)+B_{n}^{k}exp\left(-\beta_{n}^{k}y\right)\right)+\\ a_{k,k-1}\sum_{n=1}^{\infty }\left(G_{xy}^{k}{\left(\beta_{n}^{k-1}\right)}^{2}-E_{xx}^{k}{\left(k_{n}\right)}^{2}\right)sin\left(k_{n}x\right)\left(A_{n}^{k-1}exp\left(\beta_{n}^{k-1}y\right)+B_{n}^{k-1}exp\left(-\beta_{n}^{k-1}y\right)\right)+\\ \sum_{q=1}^{k-2}a_{k,q}\sum_{n=1}^{\infty }\left(G_{xy}^{k}{\left(\beta_{n}^{q}\right)}^{2}-E_{xx}^{k}{\left(k_{n}\right)}^{2}\right)sin\left(k_{n}x\right)\left(A_{n}^{q}exp\left(\beta_{n}^{q}y\right)+B_{n}^{q}exp\left(-\beta_{n}^{q}y\right)\right)\\ =\sum_{i=1}^{w}\left(1-A_{2i}^{k}\right)A_{1i}^{k-1}\sum_{n=1}^{\infty }\left(G_{xyi}{\left(\beta_{n}^{k-1}\right)}^{2}-E_{xxi}{\left(k_{n}\right)}^{2}\right)sin\left(k_{n}x\right)\left(A_{n}^{k-1}exp\left(\beta_{n}^{k-1}y\right)+B_{n}^{k-1}exp\left(-\beta_{n}^{k-1}y\right)\right)+\\ \sum_{q=1}^{k-2}\sum_{i=1}^{w}\left(1-A_{2i}^{k}\right)d_{i}^{k-1,q}\sum_{n=1}^{\infty }\left(G_{xyi}{\left(\beta_{n}^{q}\right)}^{2}-E_{xxi}{\left(k_{n}\right)}^{2}\right)sin\left(k_{n}x\right)\left(A_{n}^{q}exp\left(\beta_{n}^{q}y\right)+B_{n}^{q}exp\left(-\beta_{n}^{q}y\right)\right) \end{array}$$

where

(A2) $$d_{i}^{k-1,q}=\sum_{p=q}^{\left(\left(k-1\right)-1\right)}\prod_{m=1}^{\left(k-1\right)-p}A_{2i}^{\left(k-2\right)-\left(m-1\right)}A_{1i}^{p}a_{p,q}+A_{1i}^{k-1}a_{k-1,q}$$

Multiplying both sides of Equation (A2) by $sin\left(k_{n}x\right)$ and integrating over the beam length, Equation (A2) can be rewritten below.

(A3) $$\begin{array}{l} a_{k,k}\left(G_{xy}^{k}{\left(\beta_{n}^{k}\right)}^{2}-E_{xx}^{k}{\left(k_{n}\right)}^{2}\right)\left(A_{n}^{k}exp\left(\beta_{n}^{k}y\right)+B_{n}^{k}exp\left(-\beta_{n}^{k}y\right)\right)+\\ a_{k,k-1}\left(G_{xy}^{k}{\left(\beta_{n}^{k-1}\right)}^{2}-E_{xx}^{k}{\left(k_{n}\right)}^{2}\right)\left(A_{n}^{k-1}exp\left(\beta_{n}^{k-1}y\right)+B_{n}^{k-1}exp\left(-\beta_{n}^{k-1}y\right)\right)\\ \sum_{q=1}^{k-2}a_{k,q}\left(G_{xy}^{k}{\left(\beta_{n}^{q}\right)}^{2}-E_{xx}^{k}{\left(k_{n}\right)}^{2}\right)\left(A_{n}^{q}exp\left(\beta_{n}^{q}y\right)+B_{n}^{q}exp\left(-\beta_{n}^{q}y\right)\right)\\ =\sum_{i=1}^{w}\left(1-A_{2i}^{k}\right)A_{1i}^{k-1}\left(G_{xyi}{\left(\beta_{n}^{k-1}\right)}^{2}-E_{xxi}{\left(k_{n}\right)}^{2}\right)\left(A_{n}^{k-1}exp\left(\beta_{n}^{k-1}y\right)+B_{n}^{k-1}exp\left(-\beta_{n}^{k-1}y\right)\right)+\\ \sum_{q=1}^{k-2}\sum_{i=1}^{w}\left(1-A_{2i}^{k}\right)d_{i}^{k-1,q}\left(G_{xyi}{\left(\beta_{n}^{q}\right)}^{2}-E_{xxi}{\left(k_{n}\right)}^{2}\right)\left(A_{n}^{q}exp\left(\beta_{n}^{q}y\right)+B_{n}^{q}exp\left(-\beta_{n}^{q}y\right)\right) \end{array}$$

It can be seen from Equation (A3) that the coefficient $a_{k,q}$ is related to the characteristic parameter $\beta_{n}^{q}$. To guarantee that the Equation (A3) can be established for any position y, it can be inferred that the corresponding items including $\beta_{n}^{q}$ should be equal for both sides of Equation (A3). Therefore, the coefficient $a_{k,q}$ can be obtained as follows:

(A4) $$a_{k,q}=\left\{ \begin{array}{l} \sum_{i=1}^{w}\left(1-A_{{}_{2i}}^{k}\right)d_{i}^{k-1,q}\left(G_{xyi}-E_{xxi}/c_{q}^{2}\right)/\left(G_{xy}^{k}-E_{xx}^{k}/c_{q}^{2}\right),q<k-1\\ \sum_{i=1}^{w}\left(1-A_{{}_{2i}}^{k}\right)A_{1i}^{k-1}\left(G_{xyi}-E_{xxi}/c_{k-1}^{2}\right)/\left(G_{xy}^{k}-E_{xx}^{k}/c_{k-1}^{2}\right),q=k-1\\ 1,q=k \end{array}\right.$$

It is known that the equivalent axial elastic modulus and shear modulus is kept unchanged in the fully relaxed state, which means $\left(G_{xy}^{k}-E_{xx}^{k}/c_{q}^{2}\right)=0$. Therefore, it can be inferred that the coefficient $a_{k,q}$ is zero when $\left(G_{xy}^{k}-E_{xx}^{k}/c_{q}^{2}\right)$ equals to zero. The coefficient $a_{k,q}$ can, thus, be rewritten as:

(A5) $$a_{k,q}=\left\{ \begin{array}{l} 0,\left(G_{xy}^{k}-E_{xx}^{k}/c_{q}^{2}\right)=0\\ \sum_{i=1}^{w}\left(1-A_{{}_{2i}}^{k}\right)d_{i}^{k-1,q}\left(G_{xyi}-E_{xxi}/c_{q}^{2}\right)/\left(G_{xy}^{k}-E_{xx}^{k}/c_{q}^{2}\right),q<k-1and\left(G_{xy}^{k}-E_{xx}^{k}/c_{q}^{2}\right)\neq 0\\ \sum_{i=1}^{w}\left(1-A_{{}_{2i}}^{k}\right)A_{1i}^{k-1}\left(G_{xyi}-E_{xxi}/c_{k-1}^{2}\right)/\left(G_{xy}^{k}-E_{xx}^{k}/c_{k-1}^{2}\right),q=k-1and\left(G_{xy}^{k}-E_{xx}^{k}/c_{k-1}^{2}\right)\neq 0\\ 1,q=k \end{array}\right.$$

It can be seen from Equation (A2) that the time complexity of $d_{i}^{k-1,q}$ is $o\left(k^{2}\right)$. If $d_{i}^{k-1,q}$ is directly solved by Equation (A2), it would considerably decrease the efficiency of the proposed solution algorithm. Therefore, some simplifications are made for the solution of $d_{i}^{k-1,q}$ as follows:

(A6) $$\begin{array}{cc} d_{i}^{k-1,q} & =\sum_{p=q}^{\left(\left(k-1\right)-1\right)}\prod_{m=1}^{\left(k-1\right)-p}A_{2i}^{\left(k-2\right)-\left(m-1\right)}A_{1i}^{p}a_{p,q}+A_{1i}^{k-1}a_{k-1,q}\\ & =\sum_{p=q}^{\left(\left(k-2\right)-1\right)}\prod_{m=1}^{\left(k-1\right)-p}A_{2i}^{\left(k-2\right)-\left(m-1\right)}A_{1i}^{p}a_{p,q}+A_{2i}^{k-1}A_{1i}^{k-2}a_{k-2,q}+A_{1i}^{k-1}a_{k-1,q}\\ & =A_{2i}^{k-1}\sum_{p=q}^{\left(\left(k-2\right)-1\right)}\prod_{m=1}^{\left(k-2\right)-p}A_{2i}^{\left(k-2\right)-\left(m-1\right)}A_{1i}^{p}a_{p,q}+A_{2i}^{k-1}A_{1i}^{k-2}a_{k-2,q}+A_{1i}^{k-1}a_{k-1,q}\\ & =A_{2i}^{k-1}\left(\sum_{p=q}^{\left(\left(k-2\right)-1\right)}\prod_{m=1}^{\left(k-2\right)-p}A_{2i}^{\left(k-2\right)-\left(m-1\right)}A_{1i}^{p}a_{p,q}+A_{1i}^{k-2}a_{k-2,q}\right)+A_{1i}^{k-1}a_{k-1,q}\\ & =A_{2i}^{k-1}d_{i}^{k-2,q}+A_{1i}^{k-1}a_{k-1,q} \end{array}$$

It can be seen that the time complexity of $d_{i}^{k-1,q}$ shown as Equation (A2) has been greatly reduced when compared to Equation (24). It is meaningful for improving the efficiency of the proposed analytical solution.

## References

[1] Ersoy N., Potter K., Wisnom M.R., Clegg M.J. (2005). Development of spring-in angle during cure of a thermosetting composite. Compos. Part A.

[2] Salomi A., Garstka T., Potter K., Greco A., Maffezzoli A. (2008). Spring-in angle as molding distortion for thermoplastic matrix composite. Compos. Sci. Technol..

[3] Kappel E., Stefaniak D., Hühne C. (2013). Process distortions in prepreg manufacturing-an experimental study on CFRP L-profiles. Compos. Struct..

[4] Zhu Q., Geubelle P.H., Li M., Tucker C.L. (2001). Dimensional accuracy of thermoset composites: Simulation of process-induced residual stresses. J. Compos. Mater..

[5] Capehart T.W., Muhammad N., Kia H.G. (2007). Compensating thermoset composite panel deformation using corrective molding. J. Compos. Mater..

[6] Wucher B., Lani F., Pardoen T., Bailly C., Martiny P. (2014). Tooling geometry optimization for compensation of cure-induced distortions of a curved carbon/ epoxy C-spar. Compos. Part A.

[7] Wisnom M.R., Gigliotti M., Ersoy N., Campbell M., Potter K.D. (2006). Mechanisms generating residual stresses and distortion during manufacture of polymer matrix composite structures. Compos. Part A.

[8] Fernlund G., Rahman N., Courdji R., Bresslauer M., Poursartip A., Willden K. (2002). Experimental and numerical study of the effect of cure cycle, tool surface, geometry, and lay-up on the dimensional fidelity of auto-clave-processed composite parts. Compos. Part A.

[9] Ruiz E., Trochu F. (2005). Numerical analysis of cure temperature and internal stresses in thin and thick RTM parts. Compos. Part A.

[10] Wang X., Jia Y., Wang C. (2012). Correlation analysis of heat transfer, cure and mechanical behavior of fiber composite laminates. Polym. Polym. Compos..

[11] Sorrentino L., Polini W., Bellini C. (2014). To design the cure process of thick composite parts: Experimental and numerical results. Adv. Compos. Mater..

[12] Dong C. (2010). Process-induced deformation of composite T-stiffener structures. Compos. Struct..

[13] Gopal A.G., Adali S., Verijenko V.E. (2000). Optimal temperature profiles for minimum residual stress in the cure process of polymer composites. Compos. Struct..

[14] Nelson R.H., Cairns D.S. Prediction of Dimensional Changes in Composite Laminates during Cure. Proceedings of the 34th International ASMPE Symposium.

[15] Radford D.W., Rennick T.S. (2000). Separating sources of manufacturing distortion in laminated composites. J. Reinf. Plast. Compos..

[16] Wisnom M.R., Potter K.D., Ersoy N. (2007). Shear-lag analysis of the effect of thickness on spring-in of curved composites. J. Compos. Mater..

[17] Twigg G., Poursartip A., Fernlund G. (2004). Tool–part interaction in composites processing. Part I: Experimental investigation and analytical model. Compos. Part A.

[18] Yuan Z., Wang Y., Peng X., Wang J., Wei S. (2016). An analytical model on through-thickness stresses and distortions of composite laminates due to tool-part interaction. Compos. Part B.

[19] Arafath A., Vaziri R., Poursartip A. (2008). Closed-form solution for process-induced stresses and deformation of a composite part cured on a solid tool: Part I: Flat geometries. Compos. Part A.

[20] Arafath A., Vaziri R., Poursartip A. (2009). Closed-form solution for process-induced stresses and deformation of a composite part cured on a solid tool: Part II: Curved geometries. Compos. Part A.

[21] Arafath A. (2007). Efficient Numerical Techniques for Predicting Process-Induced Stresses and Deformations in Composite Structures. Ph.D. Thesis.

[22] Abouhamzeh M., Sinke J., Jansen K.M.B., Benedictus R. (2015). Closed form expression for residual stresses and warpage during cure of composite laminates. Compos. Struct..

[23] Ding A., Li S., Wang J., Ni A. (2017). A new analytical solution for spring-in of curved composite parts. Compos. Sci. Technol..

[24] Erik K. (2016). Forced-interaction and spring-in-Relevant initiators of process-induced distortions in composite manufacturing. Compos. Struct..

[25] Costanzo B., Luca S. (2016). Analysis of cure induced deformation of CFRP U-shaped laminates. Compos. Struct..

[26] Schapery R.A. (1969). On the characterization of nonlinear viscoelastic materials. Polym. Eng. Sci..

[27] Li J., Yao X., Liu Y., Cen Z., Kou Z., Hu X., Dai D. (2010). Thermo-viscoelastic analysis of the integrated T-shaped composite structures. Compos. Sci. Technol..

[28] Ding A., Li S., Sun J., Wang J., Zu L. (2016). A thermo-viscoelastic model of process induced residual stresses in composite structures with considering thermo dependence. Compos. Struct..

[29] Abouhamzeh M., Sinke J., Jansen K.M.B., Benedictus R. (2015). A new procedure for thermo-viscoelastic modeling of composites with general orthotropy and geometry. Compos. Struct..

[30] Abouhamzeh M., Sinke J., Jansen K.M.B., Benedictus R. (2016). Thermo-viscoelastic analysis of GLARE. Compos. Part B.

[31] Zobeiry N., Vaziri R., Poursartip A. (2010). Computationally efficient pseudo-viscoelastic models for evaluation of residual stresses in thermoset polymer composites during cure. Compos. Part A.

[32] Zobeiry N., Vaziri R., Poursartip A. (2005). Differential implementation of the viscoelastic response of a curing thermoset matrix for composites processing. J. Eng. Mater..

[33] Zobeiry N., Vaziri R., Poursartip A. (2016). A differential approach to finite element modeling of isotropic and transversely isotropic viscoelastic materials. Mech. Mater..

[34] Zhang J., Zhang M., Li S., Pavier M.J., Smith D.J. (2016). Residual stresses created during curing of a polymer matrix composite using a viscoelastic model. Compos. Sci. Technol..

[35] Kim Y.K. (1996). Process-Induced Viscoelastic Residual Stress Analysis of Graphite/Epoxy Composite Structures. Ph.D. Thesis.

[36] Timoshenko S.P. (1925). Analysis of bi-metal thermostats. J. Opt. Soc. Am. A.

[37] Robbins D.H., Reddy J.N. (1993). Modelling of thick composites using a layerwise laminate theory. Int. J. Numer. Methods Eng..

[38] Kim K., Hahn H.T. (1989). Residual stress development during processing of graphite/epoxy composites. Compos. Sci. Technol..

[39] Wijskamp S., Akkerman R., Lamers E.A.D. Residual stresses in non-symmetrical Carbon/epoxy laminates. Proceedings of the ICCM-14 Conference.

[40] Gigliotti M., Wisnom M.R., Potter K.D. (2003). Development of curvature during curing of AS4/8552 [0/90] unsymmetric composite plates. Compos. Sci. Technol..

[41] Ersoy N., Potter K., Wisnom M.R., Clegg M.J. (2005). An experimental method to study the frictional processes during composites manufacturing. Compos. Part A.

